# Bio-Inspired Sensitivity-Weighted NSGA-II Optimization of a 6-UPS Parallel Loading Mechanism for Aero-Engine Pylon Vector-Force Loading

**DOI:** 10.3390/biomimetics11070444

**Published:** 2026-06-24

**Authors:** You Zhang, Yang Pan, Lingyu Wang, Haoran Cui, Surong Jiang, Liping Ding, Shengli Chen, Yangshuo Yue, Bai Chen

**Affiliations:** 1 College of Mechanical and Electrical Engineering, Nanjing University of Aeronautics and Astronautics, Nanjing 210016, China; xiaoxiaozhang@nuaa.edu.cn (Y.Z.); cui_hr@nuaa.edu.cn (H.C.); jsrong@nuaa.edu.cn (S.J.); lpding@nuaa.edu.cn (L.D.); chenbye@nuaa.edu.cn (B.C.); 2 Science and Technology on Altitude Simulation Laboratory, Sichuan Gas Turbine Establishment, Aero Engine Corporation of China, Jiangyou 621700, China; 3 Jiangsu Product Quality Testing and Inspection Institute, No. 5 Guanghua East St., Nanjing 210007, China; chenshl@mail.ustc.edu.cn (S.C.); yue.yangshuo@jszj.net.cn (Y.Y.)

**Keywords:** bio-inspired evolutionary optimization, sensitivity-weighted NSGA-II, 6-UPS parallel mechanism, variable-wise search intensity, vector-force loading, sensitivity-driven optimization

## Abstract

Structural static testing is paramount for validating the structural integrity of critical aerospace components. However, conventional test rigs are often constrained to fixed loading axes and frequently induce parasitic torques. Accurate reproduction of aero-engine pylon flight loads therefore requires a mechanism that combines omnidirectional vector loading, high stiffness, and efficient force transmission. Achieving these coupled requirements is primarily a geometric synthesis problem, yet the associated workspace, stiffness, and load–capacity indices are nonlinear, mutually coupled, and expensive to evaluate over dense pose samples. To address this optimization bottleneck, this work develops a task-specific 6-UPS loading mechanism and a bio-inspired sensitivity-weighted NSGA-II algorithm for its geometric synthesis. Inspired by gene/locus-specific heterogeneity in biological evolution, the algorithm assigns variable-wise search intensities according to design-variable sensitivities, which are estimated using Multivariate Adaptive Regression Splines (MARS). In this way, influential design genes receive stronger local exploitation, whereas less sensitive ones retain broader exploration. Numerical simulations demonstrate that the proposed approach reduces computation time from about 30 h to 3 h relative to direct optimization with the baseline NSGA-II, while simultaneously improving workspace, stiffness, and load-carrying capacity. A hybrid physical prototype was further tested under 240 loaded pose conditions; the system maintained force magnitude errors below 0.64% (63.42 N) and directional deviations below 1.15°. These results support the efficacy of the proposed bio-inspired optimization-based design methodology for high-fidelity static testing of aero-engine pylons under the adopted hybrid setup.

## 1. Introduction

Ensuring the structural safety of an aircraft’s key components is paramount [1]. The engine pylon, serving as the primary structural interface between the aero-engine and the wing, is subjected to severe and alternating loads during flight operations. Consequently, reliability verification through rigorous structural testing is imperative [2]. These tests are usually performed as static or quasi-static loading campaigns [3]. In such tests, loads derived primarily from engine thrust and inertia are applied at the engine’s center of gravity [4]. Typical practice is to reproduce critical flight load cases (worst-case flight loading conditions) by applying prescribed three-dimensional forces to the pylon or a representative attachment. When needed, bending or torsional moments are also applied [5]. The structural response is then assessed by recording deflections, strains, and interface loads to verify the safety of the structure [6]. Critically, the validity of this verification relies on the precise reproduction of load vectors. Any deviation in the loading direction or the introduction of parasitic moments can distort the stress distribution, potentially yielding erroneous structural margin estimations. Therefore, a high-fidelity loading system is required.

In standard pylon testing scenarios, resultant engine forces dominate the load spectrum. Consequently, the application of three prescribed orthogonal forces is typically sufficient, obviating the need for independent moment inputs [5]. However, comprehensive verification mandates applying these force vectors across a broad envelope of attitudes to accurately simulate complex flight conditions. This requirement necessitates a test system capable of omnidirectional reach within a large workspace while maintaining precise force alignment [7]. Furthermore, as the pylon load path is statically indeterminate, the internal load distribution is highly sensitive to boundary compliance, making the stiffness of the loading system a critical factor for fidelity [8]. Simultaneously, the substantial magnitude of operational loads imposes stringent demands on the load-carrying capacity of the actuators. Synthesizing these constraints, the primary design objectives for the proposed system are defined as: an expansive regular workspace, high structural stiffness, and robust force transmission capability.

Conventionally, pylon static tests utilize orthogonal hydraulic actuators to deliver prescribed forces [9]. The coordination of multiple independent actuators introduces inter-channel coupling and synchronization errors, which invariably generate parasitic moments. Moreover, because the hydraulic actuators are fixed along preset axes by fixture geometry and boundary constraints, the load directions are restricted to discrete orientations. As a result, arbitrary vector loading cannot be achieved. To address these limitations, researchers have adopted parallel-robot-based multi-axis loading systems. With their structural stiffness, control precision, and high load capacity, parallel mechanisms are promising for multi-dimensional force loading. This approach has been applied to reliability testing of machine tools by imposing multi-axis loads on spindles and shafts [10,11], and to complex aero-engine components, where synchronous loading of multiple flanges improves realism and control fidelity [12]. Nevertheless, when commanding full six-component wrenches, even minor calibration errors can induce unintended torques at the specimen interface. Consequently, achieving pure, high-magnitude vector loading across arbitrary directions while structurally suppressing parasitic moments remains a challenge.

Crucially, the ability of a parallel mechanism to meet these stringent operational requirements is intrinsically determined by its geometric configuration. Therefore, maximizing performance metrics such as workspace volume, global stiffness, and load-carrying capacity requires rigorous dimensional synthesis. To address the inherent non-linearity and irregular solution space of such mechanisms, population-based metaheuristics such as NSGA-II [13,14] and Particle Swarm Optimization (PSO) [15] are typically preferred due to their robust, gradient-free global search capabilities. However, these algorithms fundamentally rely on large-scale iterative evaluations. When coupled with the computationally intensive process of calculating global performance indices via dense sampling, their direct application becomes prohibitively time-consuming. To resolve this efficiency conflict, surrogate models are frequently employed to map design variables to fitness functions [16]. Common approaches utilize Gaussian Process Regression [17], Radial Basis Function (RBF) models [18], and Artificial Neural Networks (ANNs) [19]. While effective at approximation, these “black box” models often lack interpretability. The opaque relationship between geometric parameters and system performance obscures engineering insight, hindering effective trade-off analysis [20]. Therefore, an ideal methodology must be both computationally efficient and physically interpretable.

To address these challenges, this work develops a task-specific 6-UPS loading system and optimizes its geometry using a bio-inspired, interpretable sensitivity-weighted NSGA-II algorithm. The loading system uses a central rod and a specimen-side spherical joint to constrain the transmitted load to axial tension-compression, enabling arbitrary vector loading while structurally decoupling parasitic torques. The algorithm is inspired by heterogeneous genetic variation in biological evolution, where different genes or loci tolerate different levels of variation because mutation rates vary across genomes and structurally or functionally constrained protein sites evolve more slowly than less critical sites [21,22]. Here, the biological inspiration is used to describe heterogeneous variation among design variables: geometric parameters with different effects on mechanism performance should not receive the same search intensity during evolutionary optimization. In the proposed optimization, each geometric variable is therefore treated as a design gene, and its allowable search intensity is determined by its sensitivity to mechanism performance. MARS-based sensitivity analysis is used only to estimate these sensitivities, which are then converted into variable-wise distribution factors in NSGA-II. As a result, sensitive design genes receive stronger local exploitation, whereas less sensitive ones retain broader exploration. Through this biologically inspired heterogeneous search, the mechanism geometry is synthesized with respect to regular-workspace size, task-direction stiffness, and load-carrying capacity.

Therefore, the main contributions of this paper are as follows:

1. Development of a task-specific 6-UPS vector-force loading mechanism:A mechanism configuration with a central rod and specimen-side spherical joint is designed to transmit forces solely through axial tension–compression, thereby structurally decoupling parasitic torques to ensure accurate vector loading.
2. Establishment of a bio-inspired sensitivity-weighted NSGA-II framework: Using MARS to estimate global design-variable sensitivities, the framework assigns variable-wise search intensities in a manner analogous to locus-specific heterogeneity in biological evolution, thereby achieving about a 90% reduction in computation time while improving workspace, stiffness, and load-carrying capacity.
3. Prototype-level experimental validation under loaded poses: Physical testing on a hybrid platform verifies the optimized configuration under 240 quasi-static loaded pose conditions, with force magnitude errors maintained below 0.64% and directional deviations below 1.15°.

The remainder of this paper is organized as follows: Section 2 details the design concept, theoretical modeling, and performance indices of the 6-UPS loading system. Section 3 presents the bio-inspired sensitivity-weighted NSGA-II optimization methodology and analyzes the optimization results. Section 4 describes the experimental validation on a physical prototype. Section 5 and Section 6 provide the discussion and conclusions, respectively.

## 2. Design and Modeling of the Loading System

### 2.1. Design Concept and Configuration

To ensure high-fidelity vector force application, the loading system is designed to maximize force capacity while strictly minimizing parasitic torques. Although traditional 6-DOF parallel mechanisms can apply both forces and torques, this generality can compromise the fidelity of pure vector-force loading. Accordingly, this study proposes a task-specific 6-UPS (Universal-Prismatic-Spherical) architecture for applying vector forces from arbitrary orientations while suppressing parasitic torque transfer.

**Figure 1:**
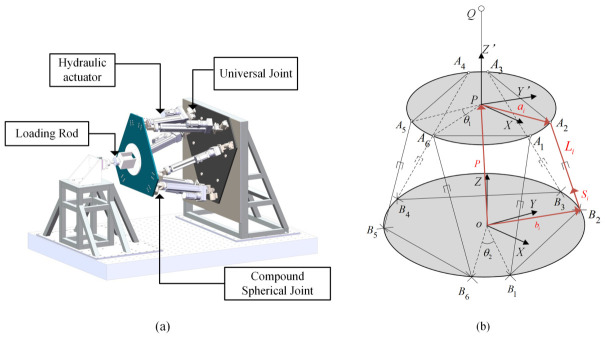
(**a**) Force-loading Stewart–Gough configuration. (**b**) Mechanism geometry diagram.

As illustrated in Figure 1a, the system consists of a fixed base and a moving platform interconnected by six identical UPS kinematic chains. Each chain includes a universal joint (U) at the base, an actuated prismatic joint (P), and a spherical joint (S) at the moving platform. To accommodate the requisite angular range without mechanical interference, the S-joints are implemented as compound spherical joints. A critical feature of this design is the central loading rod, rigidly mounted to the moving platform via a flange. This rod connects to the test specimen through an additional spherical joint, strictly constraining the load path to axial tension-compression. Hydraulic actuators drive the prismatic joints, with integrated uniaxial force sensors providing real-time feedback for closed-loop control. This configuration ensures that all applied forces are transmitted directly along the loading rod, effectively eliminating parasitic torques at the specimen interface.

### 2.2. Kinematic and Static Force Analysis

A rigorous evaluation of performance indices necessitates a comprehensive kinematic and static model of the mechanism. The geometry of the 6-UPS mechanism is depicted in Figure 1b. The spherical joint attachment points on the moving platform are denoted as $A_{i}$ (i=1,…,6), and the universal joint attachment points on the base are denoted as $B_{i}$ (i=1,…,6). Sets $A_{i}$ and $B_{i}$ lie on circles of radius *r* and *R* on their respective platforms and are arranged with threefold rotational symmetry; their in-plane angular locations are $\theta_{1}$ (moving platform) and $\theta_{2}$ (base). The geometric center of the spherical joint at the end of the loading rod is denoted as *Q*.

Based on this geometry, two coordinate frames are established: The fixed frame O{x,y,z} has origin *O* at the geometric center of the base platform. The *z*-axis is perpendicular to the base plane, and the *x*-axis is directed towards the midpoint of joint connection points $B_{1}$ and $B_{2}$. The moving frame $P\{x^{\prime },y^{\prime },z^{\prime }\}$ has origin *P* at the geometric center of the moving platform; The $z^{\prime }$-axis is perpendicular to the moving platform plane, and the $x^{\prime }$-axis is directed towards the midpoint of joint connection points $A_{1}$ and $A_{2}$.

#### 2.2.1. Inverse Kinematics

Then the kinematic model is established from the vector-loop closure shown in Figure 1b. Let p be the position vector from origin *O* to origin *P*. Let biO be the position vector of the joint $B_{i}$ in the fixed frame, and let aiP be the position vector of the joint $A_{i}$ in the moving frame. The leg vector, extending from $B_{i}$ to $A_{i}$, is denoted as $l_{i}=l_{i}s_{i}$, where $l_{i}$ is the instantaneous leg length and $s_{i}$ is its unit direction vector. The platform pose is denoted by $q={\left[p,\theta \right]}^{T}$, where θ=[α,β,γ] and p=[x,y,z]. Here, a ZXZ Euler convention is adopted for θ. Therefore, the vector-loop closure equation can be expressed as:

(1) RPO(θ)aiP+p=lisi+biO

With the adopted convention, RPO(θ)=Rz(α)Rx(β)Rz(γ) is written explicitly as

(2) RPO(θ)=cαcγ−sαcβsγ−cαsγ−sαcβcγsαsβsαcγ+cαcβsγ−sαsγ+cαcβcγ−cαsβsβsγsβcγcβ

where c(·) and s(·) denote cosine and sine, respectively. Therefore, the length of each leg can be calculated from the platform pose by taking the magnitude of both sides of Equation (1):

(3) li=|RPOaiP−biO+p|

#### 2.2.2. Static Force Analysis

The relationship between the actuator forces along the legs and the external wrench applied on the moving platform is derived from the principle of virtual work. Under static equilibrium, and neglecting friction and gravity, the virtual work of the actuators equals that of the external wrench:

(4) $$W^{T}\delta q=F^{T}\delta l$$

Here, $F={\left[F_{1},F_{2},F_{3},F_{4},F_{5},F_{6}\right]}^{T}$ denotes the vector of actuator forces, and $W={\left[f^{T},\tau^{T}\right]}^{T}$ is the external wrench (force f and torque τ) applied to the moving platform. Let $\delta l\in R^{6}$ be the vector of virtual leg-length variations, and $\delta q={\left[\delta p^{T},\delta \theta^{T}\right]}^{T}\in R^{6}$ is the vector of virtual platform displacements (the twist). For the *i*th limb, taking the first-order differential of Equation (1) yields

(5) δp+δ(RPOaiP)=δlisi+liδsi

Premultiplying both sides by $s_{i}^{T}$ and using the orthogonality condition $s_{i}^{T}\delta s_{i}=0$ gives

(6) δli=siTδp+siTδ(RPOaiP)

For an infinitesimal platform rotation, the rotational variation term can be expressed as

(7) δ(RPOaiP)=δθ×(RPOaiP)

Therefore, the virtual length variation of each limb is

(8) δli=siTδp+RPOaiP×siTδθ

Stacking the six limb equations leads to

(9) δl=s1T(RPOa1P×s1)T⋮⋮s6T(RPOa6P×s6)Tδpδθ=Jδq

where J is the Jacobian matrix.

Substituting this result back into the virtual work Equation (4) gives:

(10) $$F^{T}\left(J\delta q\right)=W^{T}\delta q\Rightarrow \left(F^{T}J-W^{T}\right)\delta q=0$$

Since this equation must hold for any arbitrary non-zero virtual displacement δq, the term in parentheses must be zero. This yields the fundamental static force mapping for the parallel mechanism:

(11) $$W=J^{T}F$$

### 2.3. Performance Analysis and Evaluation

To guide the dimensional synthesis, quantitative performance indices for workspace, stiffness, and load-carrying capacity are formulated.

#### 2.3.1. Workspace

The workspace is a key performance metric that specifies the range of motion available to the end-effector [23]. In this work, the Reachable Workspace $W_{\mathrm{reach}}$ is defined as the set of poses that can be achieved without violating geometric or joint limits. This set can be formally expressed as:

(12) $$\begin{array}{cc} \; & W_{\mathrm{reach}}=\left\{\left(p,\theta \right)|p\left(\theta \right)=\left(x,y,z,\alpha ,\beta ,\gamma \right)\right\}\\ \; & s.t.\;\left\{\begin{array}{c} L_{\mathrm{rod}}=\sqrt{{\left(x-x_{c}\right)}^{2}+{\left(y-y_{c}\right)}^{2}+{\left(z-z_{c}\right)}^{2}}\\ l_{\min}\leq l_{i}\leq l_{\max}\;\left(i=1,\ldots ,6\right)\\ \theta_{\mathrm{joint},i}\leq \theta_{\mathrm{joint},\max}\;\left(i=1,\ldots ,6\right) \end{array}\right. \end{array}$$

To determine the reachable workspace based on the boundary conditions in Equation (12), a discrete search method is applied in Cartesian space. The target spatial envelope is first discretized into a grid of specific poses. For each pose, the required leg lengths are computed using the inverse kinematic model (Equation (1)). These computed lengths are then evaluated against the physical limits established in Equation (12). Poses satisfying all mechanical constraints constitute the valid reachable workspace. This direct Cartesian-to-joint mapping effectively evaluates the mechanism’s kinematic feasibility.

**Figure 2:**
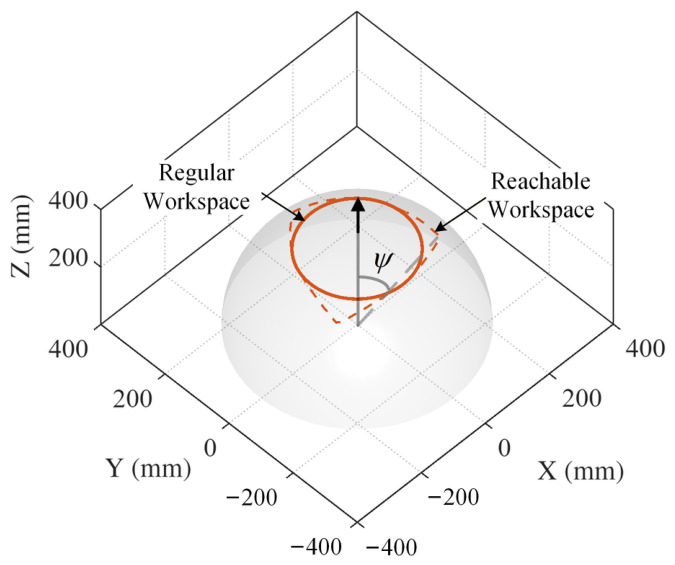
Reachable workspace (Dashed Line) and regular workspace (Solid Line) of the manipulator.

The first condition ensures motion on a spherical surface defined by the loading rod length $L_{\mathrm{rod}}$, while the subsequent conditions represent the actuator stroke limits and joint angular limits, respectively. Accordingly, we parameterize poses on the spherical surface by the polar angle ψ and an azimuthal angle ϕ to represent the point in the workspace. During workspace evaluation, poses associated with a rank-deficient or numerically near-singular Jacobian are excluded; for the present 6-UPS mechanism, this corresponds to Type-II singular configurations in which J loses rank. In implementation, this is checked through the rank of J together with its smallest singular value. These constraints yield a reachable set with a complex, irregular boundary, which is inconvenient as a direct objective in optimization. Therefore, we introduce a regular workspace $W_{\mathrm{reg}}$ defined as the largest spherical cap fully inscribed within $W_{\mathrm{reach}}$. As illustrated in Figure 2, the resulting regular workspace represents a singularity-free operating region within the prescribed loading envelope.

(13) $$W_{\mathrm{reg}}\left(\psi_{\min}\right)=\left\{\left(\psi ,\phi \right):0\leq \psi \leq \psi_{\min},0\leq \phi \leq 2\pi \right\}\subseteq W_{\mathrm{reach}}$$

Its size is monotonically determined by the polar angle $\psi_{\min}$ (the surface area is $A\left(\psi_{\min}\right)=2\pi L_{\mathrm{rod}}^{2}\left(1-\cos\psi_{\min}\right)$). For optimization efficiency, we use $\psi_{\min}$ as a single representative parameter. Therefore, the Workspace Index (WSI) is defined simply by the polar angle $\psi_{\min}$ of this largest spherical cap:

(14) $$\mathrm{WSI}=\psi_{\min}$$

This metric provides a scalar representation of the effective omnidirectional range of motion. For the present mechanism, the regular workspace is defined as a spherical-cap region on a sphere of fixed radius $L_{\mathrm{rod}}$. Therefore, its size is monotonically determined by the polar angle $\psi_{\min}$, making the WSI a compact and sufficient descriptor for this task-specific workspace, rather than a generic substitute for arbitrary volumetric workspace metrics.

#### 2.3.2. Stiffness

Stiffness quantifies the mechanism’s ability to resist deformation under an external wrench and is a crucial factor for ensuring the accuracy of load application [24]. In this work, the moving platform and joints are assumed to be rigid bodies, with compliance originating primarily from the axial deformation of the legs. This simplification is adopted to obtain a computationally efficient analytical stiffness model for geometry evaluation and comparative optimization, rather than a full structural compliance model of the complete prototype.

The relationship between an external wrench W applied to the moving platform and the resulting infinitesimal displacement δq is described by the overall stiffness matrix $K_{\mathrm{world}}$ in the fixed frame {O}:

(15) $$W=K_{\mathrm{world}}\delta q$$

This stiffness matrix is derived from the stiffness of the individual legs $K_{\mathrm{leg}}=\mathrm{diag}\left(k_{1},\ldots ,k_{6}\right)$, where $k_{i}=E_{i}A_{i}/l_{i}$ is the axial stiffness of the *i*-th leg. Accordingly, the stiffness model in Equation (15) is used in this work as an optimization-stage analytical approximation. It captures the dominant effect of leg axial stiffness on the overall Cartesian stiffness, but does not explicitly include joint compliance, friction, or local structural deformation. More complete elastostatic models that include link and joint compliance are available in the literature [25,26], but their use inside the dense workspace-sampling loop would substantially increase the optimization cost. Consistent with this trade-off, optimization-stage studies of parallel manipulators often employ simplified analytical stiffness models or stiffness-related indices for comparative geometry synthesis [27,28]. Using the static model developed above, the overall stiffness matrix $K_{\mathrm{world}}$ in the fixed frame is assembled from leg axial stiffness and the Jacobian:

(16) $$K_{\mathrm{world}}={\left(J\left(\frac{1}{E_{L}A_{L}}\mathrm{diag}\left(l_{1},\ldots ,l_{6}\right)\right)J^{T}\right)}^{-1}$$

Because both J=J(q) and $l_{i}=l_{i}\left(q\right)$ depend on the platform pose, Equation (15) is evaluated pointwise at every sampled pose in the workspace rather than only at q=0. Thus, the resulting stiffness map explicitly accounts for the variation of geometric transmission characteristics throughout the loading envelope.

For this application, stiffness along the loading axis ($z^{\prime }$-axis) is paramount. Therefore, to evaluate this task-specific stiffness, the global stiffness matrix $K_{\mathrm{world}}$ was transformed from the fixed frame {O} to the moving frame {P}. This is achieved using a rotation transformation matrix H=diag(RPO,RPO)

(17) $$\begin{array}{ccc} \; & HW=\left(HK_{\mathrm{world}}H^{T}\right)\left(H\delta q\right) & \end{array}$$

(18) $$\begin{array}{cc} \; & K_{\mathrm{local}}=HK_{\mathrm{world}}H^{T} \end{array}$$

The resulting $K_{\mathrm{local}}$ represents the stiffness relative to the moving platform’s coordinate system. From this matrix, we can define a Local Stiffness Index (LSI), which directly measures the stiffness along the axis of force application (the $z^{\prime }$-axis). The LSI is defined as the third diagonal element of the local stiffness matrix:

(19) $$\mathrm{LSI}=K_{\mathrm{local},33}$$

While the LSI provides a measure of stiffness at a specific pose, it varies throughout the workspace. To obtain a single, comprehensive metric for multi-objective optimization, a Global Stiffness Index (GSI) is required. Since the stiffness is first transformed into the moving-platform frame, the LSI already measures the task-direction stiffness along the local loading axis. In the present design problem, no additional preference is prescribed among admissible poses, so the GSI is defined as the uniform average of LSI over the regular workspace [25]:

(20) $$\mathrm{GSI}=\frac{\int_{V_{r}}\mathrm{LSI}dV_{r}}{\int_{V_{r}}dV_{r}}\;\mathrm{or}\;\mathrm{GSI}=\frac{1}{n}\sum_{i=1}^{n}{\mathrm{LSI}}_{i},$$

#### 2.3.3. Load-Carrying Capacity

The load-carrying capacity determines the maximum external wrench the mechanism can generate. For a parallel mechanism, the overall capacity is limited by the actuator that experiences the peak load; any imbalance in force distribution can cause early saturation and degrade system performance [29,30]. Therefore, optimizing for a balanced force distribution is essential.

The required actuation force vector, F, for a given external wrench, W, applied at the moving platform can be calculated from the inverse of the static model:

(21) F=(JT)−1RPOWp

To enable fair comparison across designs, we evaluate load-carrying capacity by applying a constant, representative test wrench and analyzing the induced actuator forces. For this application, a pure force of magnitude *f* is applied along the local $z^{\prime }$-axis of the moving platform, so the wrench in the moving frame is $W_{p}={\left(0,0,f,0,0,0\right)}^{T}$.

The performance at a specific pose is quantified by the Local Load-Carrying Capacity Index (LLI). This index is defined as the ratio of the magnitude of the applied external force to the maximum absolute actuation force required among the six legs. A higher LLI indicates a more efficient force transmission.

(22) $$\mathrm{LLI}=\frac{∥W_{p}∥}{\max(|F_{i}|)}$$

To evaluate the mechanism’s overall performance, the Global Load-Carrying Capacity Index (GLI) is defined. The global index (GLI) is the minimum LLI over the regular workspace, and the optimization aims to maximize GLI to ensure low and well-balanced actuation forces throughout the operating range.

(23) $$\mathrm{GLI}=\min_{q\in W_{\mathrm{reg}}}\left(\mathrm{LLI}\left(q\right)\right)$$

### 2.4. Mechanism-Level Advantage Analysis

**Figure 3:**
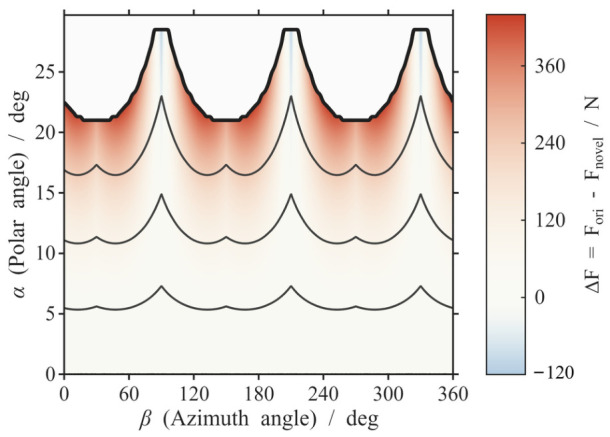
Heat map of the reduction in maximum actuator force achieved by the proposed posture-adjusting setup relative to a conventional fixed-posture Stewart platform over the sampled attitude plane (α,β).

To substantiate the structural superiority of the proposed design before dimensional optimization, we conducted a comparative static analysis using the established models. The proposed posture-adjusting setup was compared against a conventional fixed-posture Stewart platform under identical 3D external vector loads. Figure 3 illustrates the reduction in maximum actuator force achieved by the proposed setup across various posture angles (α and β).

The results demonstrate a clear mechanism-level advantage. The proposed setup (red surface) consistently demands less maximum actuator force than the conventional setup (blue surface). As highlighted by the difference plot, the posture-adjusting strategy reduces the peak actuator effort by up to 400 N in certain workspace regions. This significant reduction in peak force indicates a more efficient internal force distribution. It quantitatively shows that, by structurally decoupling the parasitic torques via the spherical joint and central rod, the proposed architecture possesses a stronger task-oriented load-carrying advantage for heavy-duty aero-engine testing.

## 3. Bio-Inspired Sensitivity-Weighted NSGA-II Optimization Methodology

To achieve the design requirements established in Section 2, the dimensional synthesis is formulated as a Multi-Objective Optimization Problem (MOP). The primary objective is to identify a geometric parameter set that simultaneously maximizes workspace volume, structural stiffness, and load transmission efficiency. To address the computationally prohibitive cost of evaluating global indices over an irregular workspace, we propose a bio-inspired sensitivity-weighted NSGA-II framework. The framework implements a heterogeneous search strategy analogous to locus-specific variation in biological evolution: design variables with different performance sensitivities are assigned different search intensities. MARS is used as an interpretable sensitivity-estimation tool, and the estimated sensitivities are then embedded into the distribution factors of the NSGA-II crossover and mutation operators.

### 3.1. Optimization Problem Formulation

To identify a design vector x that simultaneously maximizes the workspace index (WSI), global stiffness (GSI), and global load-carrying capacity (GLI), the design vector is established as $x={\left[L_{\mathrm{rod}},\theta_{1},\theta_{2},r/R,\delta_{l}\right]}^{T}$. This vector comprises five fundamental geometric parameters: the loading rod length $L_{\mathrm{rod}}$, the joint distribution angles on the moving and base platforms ($\theta_{1},\theta_{2}$), the platform radius ratio r/R, and the actuator stroke range $\delta_{l}$.

The optimization problem is rigorously defined to maximize the objective vector F(x) subject to geometric feasibility bounds and functional workspace requirements:

(24) $$\begin{array}{cc} \mathrm{Find}\; & x={\left[L_{\mathrm{rod}},\theta_{1},\theta_{2},r/R,\delta_{l}\right]}^{T}\\ \mathrm{To}\;\mathrm{maximize}\; & F\left(x\right)={\left[\mathrm{WSI}\left(x\right),\mathrm{GSI}\left(x\right),\mathrm{GLI}\left(x\right)\right]}^{T}\\ \mathrm{Subject}\;\mathrm{to}:\; & 300\;\mathrm{mm}\leq L_{\mathrm{rod}}\leq 800\;\mathrm{mm}\\ \; & 15{}^{\circ}\leq \theta_{1}\leq 40{}^{\circ}\\ \; & 15{}^{\circ}\leq \theta_{2}\leq 40{}^{\circ}\\ \; & 0.5\leq r/R\leq 1.0\\ \; & 300\;\mathrm{mm}\leq \delta_{l}\leq 500\;\mathrm{mm}\\ \; & \mathrm{WSI}\left(x\right)\geq 25{}^{\circ} \end{array}$$

The specified box constraints are strictly delimited by manufacturing capabilities and spatial assembly limitations, ensuring the physical realizability of the mechanism [31]. Furthermore, the functional constraint WSI(x)≥25° is imposed to satisfy the specific operational requirements for aero-engine thrust vectoring. This minimum angular range guarantees that the test rig can reproduce the full envelope of critical flight attitudes without encountering kinematic singularities or boundary violations.

### 3.2. Surrogate-Assisted Optimization Framework

**Figure 4:**
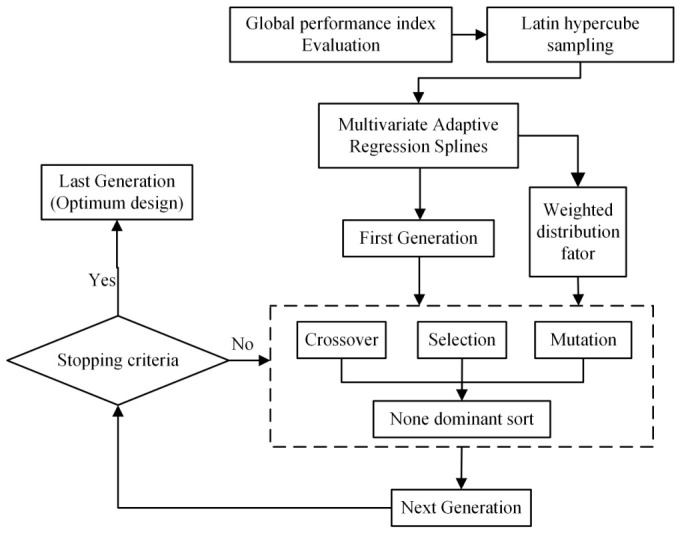
Flowchart of the proposed interpretable, sensitivity-driven multi-objective optimization framework.

Direct optimization using the full theoretical model is computationally intractable due to the dense sampling required for global index evaluation. To resolve this, a three-stage framework is implemented. The complete procedure of the framework is illustrated in Figure 4. It integrates surrogate modeling, sensitivity analysis, and evolutionary optimization into a cohesive procedure. The key stages are as follows:

1. Data Sampling and Surrogate Modeling: A one-time set of 1000 training samples is generated via Latin Hypercube Sampling (LHS) to build an efficient MARS surrogate model, which drastically reduces the computational burden from the tens of thousands of evaluations required for direct optimization.
2. Sensitivity Analysis and Search Enhancement: The interpretability of the trained MARS model is exploited to conduct a sensitivity analysis. This analysis quantifies the influence of each design parameter on the performance indices, and the results are used to design a weighted distribution factor to enhance the search efficiency of the genetic algorithm.
3. Multi-Objective Optimization: The NSGA-II algorithm, guided by the surrogate model and the weighted distribution factor, performs the multi-objective search to find the set of Pareto-optimal solutions.

The subsequent subsections will provide a detailed description of each component within this framework.

#### 3.2.1. Surrogate Modeling with MARS

To implement the proposed framework, MARS was selected to build the approximation models for the WSI, GSI, and GLI performance indices. MARS is a nonparametric regression model that also yields variable-importance measures [32,33,34], which makes it well suited to fitting the performance map of parallel mechanisms with strong nonlinear couplings and to extracting parameter sensitivities for structural design.

The MARS model approximates a function $\hat{f}\left(x\right)$ by constructing a linear combination of basis functions (BFs). The general form of the model is:

(25) $$\hat{f}\left(x\right)=\beta_{0}+\sum_{m=1}^{M}\beta_{m}h_{m}\left(x\right)$$

where $\beta_{0}$ is the constant intercept, *M* is the number of BFs, and $\beta_{m}$ are the coefficients estimated using the least squares method. A key strength of MARS is its ability to capture interaction effects between variables, as each basis function $h_{m}\left(x\right)$ can be a product of one or more hinge functions:

(26) $$h_{m}\left(x\right)=\prod_{k=1}^{K_{m}}{\left[s_{km}\cdot \left(x_{v(k,m)}-t_{km}\right)\right]}_{+}$$

Here, $K_{m}$ is the number of hinge functions in the product, ${\left[\cdot \right]}_{+}$ is the hinge function (returning its argument if positive, zero otherwise), $x_{v(k,m)}$ is the predictor variable, $t_{km}$ is the knot location on that variable, and $s_{km}=\pm 1$. This structure, where the model automatically selects variables, knots, and interactions from the data, provides the flexibility and interpretability essential for this work.

The construction of the MARS surrogates began with the generation of a comprehensive training dataset. All timing results are wall-clock measurements obtained in MATLAB R2025a on a workstation with an Intel Core Ultra 7 265KF processor and 24 GB RAM. Direct full-model NSGA-II required about 30 h for the present population and generation settings. The surrogate-assisted workflow required about 3 h, including the one-time Latin hypercube sampling of 1000 design points, full-model evaluation of these samples, and MARS model construction. Given the five-dimensional design space and the smooth, continuous nature of the expected responses, this sample size is sufficient to capture the primary trends and nonlinearities needed for a high-fidelity surrogate.

The predictive accuracy of the trained models was validated with a two-stage procedure to assess both internal consistency and generalization. First, a 10-fold cross-validation procedure was performed on the original 1000-point training dataset. The results in Table 1 confirm the high fidelity and consistency of the surrogates. The coefficients of determination ($R^{2}$) are exceptionally high for all three models (GLI: 0.998, WSI: 0.995, GSI: 0.998), indicating an almost perfect correlation between predicted and actual values. The error metrics are also small. In particular, the Normalized Root Mean Square Error (NRMSE) is less than 1.5% for all models, showing that prediction errors are small in magnitude. The low Generalized Cross-Validation (GCV) scores further indicate that this accuracy is achieved without overfitting the training data.

**Figure 5:**
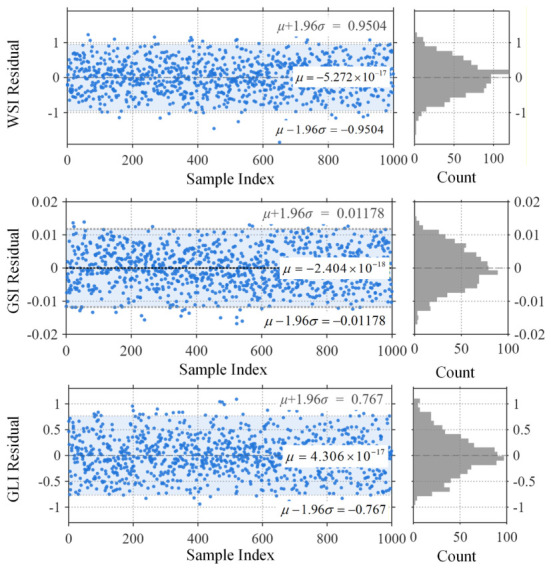
Residual distributions of the MARS surrogate model for WSI, GSI, and GLI on the 1000-point independent test set. The scatter plots show the residuals for each sample together with the mean and the μ±1.96σ bounds, while the accompanying histograms illustrate the approximately unbiased and homoscedastic error behaviour of the surrogate.

Second, an independent holdout test set of 1000 additional samples was employed. A residual analysis was carried out for the three response models, as shown in Figure 5. In each case, the residuals are tightly clustered around zero and randomly scattered over the sample index, with no visible trend over the prediction range. The empirical mean of the residuals is essentially zero, and almost all points fall within the μ±1.96σ confidence bounds, which indicates small and approximately homoscedastic errors. The accompanying histograms are nearly symmetric and bell-shaped, suggesting no systematic bias. Taken together, these observations show that the trained MARS models provide accurate, unbiased, and robust surrogate representations of the underlying system, suitable for the subsequent multi-objective optimization across the design space.

#### 3.2.2. Sensitivity Analysis and Search Enhancement

**Figure 6:**
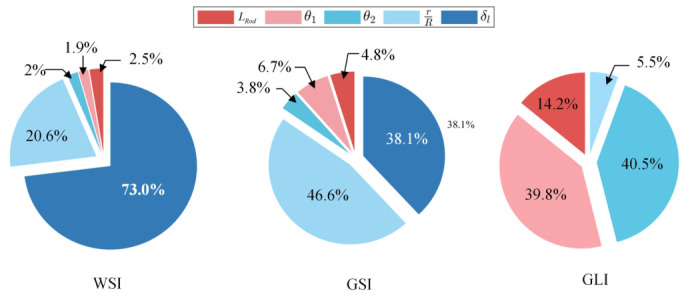
MARS-based sensitivity analysis of the three performance indices (WSI, GSI, and GLI), showing the relative contribution of leg stroke, radius ratio, and joint angles to each index.

MARS facilitates the explicit extraction of global sensitivity indices based on the reduction in generalized GCV error. The results are visualized in Figure 6. This data-driven insight is strongly supported by the physical principles of the mechanism’s kinematics and statics.

The analysis reveals that the Workspace (WSI) is dominated by the leg stroke $\delta_{l}$, which is physically intuitive: the available extension directly bounds the reachable region. The radius ratio r/R defines the mechanism’s “aspect ratio”; a smaller one results in more inclined legs, which provides greater geometric clearance for angular motions. Similarly, the overall Stiffness (GSI) is shown to be primarily governed by a combination of the radius ratio and the actuator stroke range ($\delta_{l}$), which is consistent with the concept of a structural ‘stance’. Finally, the Load-Carrying Capacity (GLI) is most sensitive to the hinge distribution angles ($\theta_{1}$ and $\theta_{2}$) as these angles directly govern the force transmission from the actuators to the end-effector.

To accelerate convergence, we introduce a bio-inspired sensitivity-weighted distribution strategy. In biological evolution, different genes and loci may exhibit different variation rates under functional constraints [21,22]. By analogy, the five geometric variables are treated as design genes in a design genotype, and WSI, GSI, and GLI are treated as performance phenotypes. The MARS sensitivity score quantifies the importance of each design gene and is converted into the variable-wise distribution factor $\eta_{i}$ in NSGA-II. Thus, sensitive variables receive more localized exploitation, while less sensitive variables retain broader exploration.

A normalized weight vector $w={\left[w_{1},\ldots ,w_{5}\right]}^{T}$ is first calculated from the sensitivity vector m. The variable-wise distribution factor is then defined as η=wc, where *c* maps the normalized sensitivity weights to the NSGA-II distribution-index scale. In this study, c=20, following the distribution-index setting used in the original real-coded NSGA-II study [13]. This keeps the baseline distribution-index scale while redistributing search intensity according to the MARS sensitivities.

(27) $$w=m/\sum_{i=1}^{5}m_{i}\;\mathrm{and}\;\eta =w\cdot c$$

**Figure 7:**
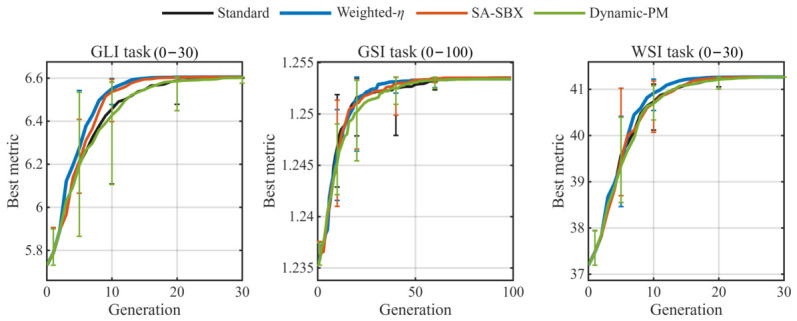
Comparison of the convergence histories under three single-objective tasks using the standard baseline, the proposed weighted-η strategy, and two representative adaptive operator schemes (SA-SBX and Dynamic-PM). The solid lines denote the mean best-metric trajectories over multiple random seeds, and the error bars indicate the corresponding min–max ranges at selected generations. Different generation windows are used for different tasks to better visualize the effective convergence stage. Overall, the proposed weighted-η strategy shows faster convergence and generally better final performance across the three tasks.

To evaluate the effect of the proposed variable-wise weighted-η strategy at the single-objective level, additional comparisons were conducted against the standard baseline and two representative adaptive operator schemes, namely SA-SBX [35] and Dynamic-PM [36]. Their convergence histories for the GLI-, GSI-, and WSI-oriented tasks are shown in Figure 7. For each task, 10 randomly selected seeds were used, and all compared methods were evaluated under the same set of seeds to ensure a fair comparison. Because the three objective functions exhibit different saturation behavior and different sensitivity to stochastic search, the population size was selected separately for each task. In addition, shortened generation windows are used for the GLI- and WSI-oriented tasks because both objectives reach convergence much earlier than the GSI-oriented task.

As shown in Figure 7, the proposed weighted-η strategy is consistently competitive across the three single-objective tasks and generally outperforms the standard baseline. It converges faster in the effective search stage and reaches higher or comparable final metric values for GLI, GSI, and WSI. The error bars also indicate acceptable run-to-run robustness: the proposed method can reach better elite solutions without a marked expansion of late-stage dispersion, whereas Dynamic-PM shows wider inter-seed ranges and weaker stability in several cases.

**Figure A1:**
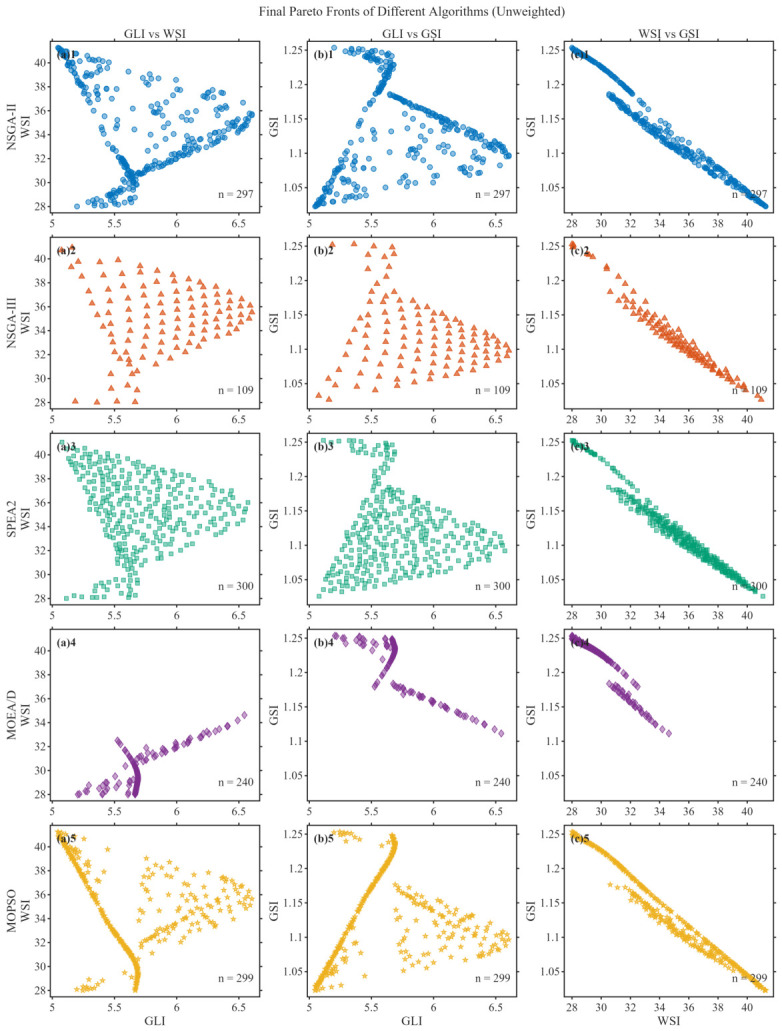
Supplementary pairwise Pareto-front projections for the proposed NSGA-II-based framework and representative multi-objective optimizers. Panels (**a1**–**a5**), (**b1**–**b5**), and (**c1**–**c5**) show the GLI–WSI, GLI–GSI, and WSI–GSI projections, respectively. Rows 1–5 correspond to NSGA-II, NSGA-III, SPEA2, MOEA/D, and MOPSO, respectively; the in-panel annotation n denotes the number of final non-dominated solutions in each row.

These results suggest that the advantage of the proposed method comes from problem-informed variable-wise weighting rather than generic adaptivity alone. Because the dominant geometric drivers differ among WSI, GSI, and GLI, a single sensitivity-weighting choice cannot be assumed a priori to be optimal for all objectives. The following multi-objective comparison therefore examines GSI-based, WSI-based, GLI-based, and unweighted NSGA-II variants, while broader comparisons with representative multi-objective optimizers are provided in Appendix A Figure A1.

### 3.3. Multi-Objective Optimization and Results

After the development of the surrogate models and the variable-wise weighting strategy, the proposed algorithm was applied to solve the multi-objective optimization problem formulated in Section 3. The algorithm was configured with a population size of 200 and was run for 100 generations. The SBX and PM operators were employed together with variable-wise weighted distribution indices, and four weighting formulations were examined in the multi-objective stage, namely GSI-based, WSI-based, GLI-based, and unweighted schemes.

**Figure 8:**
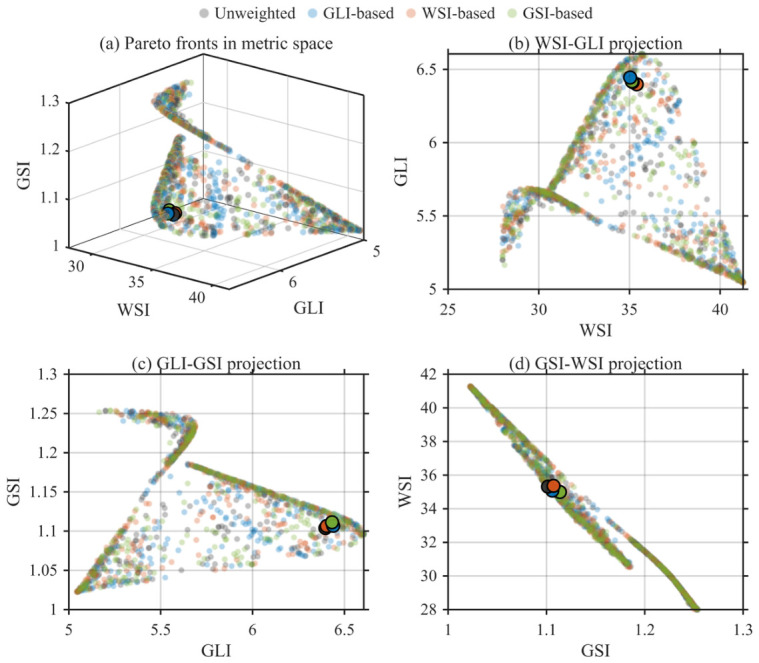
Comparison of the final non-dominated solution sets obtained under different weighting schemes in the three-objective space. The overall Pareto boundaries are broadly similar for the GSI-based, WSI-based, GLI-based, and unweighted formulations, indicating that the final attainable trade-off surface is not strongly affected by the specific weighting target. The large filled markers denote the mean values of the top-50 non-dominated subset for each scheme, which reveal slight differences in the distribution tendency of the high-quality solutions.

The optimization process yielded a set of non-dominated solutions, known as the Pareto front. Each point on this front represents an optimal trade-off, where improving one performance objective necessitates a compromise in at least one other. To further assess the influence of weighting choice, the Pareto fronts obtained from the four schemes are compared in Figure 8, and the corresponding top-50 non-dominated-subset mean values are listed in Table 2. As shown in Figure 8, the final non-dominated solution sets obtained from the four schemes occupy highly similar regions in the three-objective space, and no obvious separation of the Pareto boundaries can be observed. This indicates that the final attainable trade-off surface is largely robust to the weighting choice under the present optimization setting. The top-50 statistics in Table 2 show that the different weighting targets mainly shift the distribution tendency of the elite solutions: the WSI-based scheme gives the highest top-50 mean value in WSI, the GSI-based scheme gives the highest top-50 mean value in GSI, and the GLI-based scheme gives the highest top-50 mean value in GLI. Therefore, the weighting choice mainly affects which part of the final Pareto set is emphasized, rather than producing a fundamentally different Pareto boundary. Considering that force-loading accuracy is the primary task requirement of the present mechanism, the GSI-based scheme was finally adopted in the subsequent design selection and discussion as a task-prioritized weighting setting.

The broader significance of introducing variable-wise weighting can be examined through the normalized Pareto-quality indicators summarized in Table 3, which also includes representative reference optimizers. Here, HV reflects the covered extent of the obtained non-dominated set, GD reflects its proximity to the reference Pareto front, IGD reflects the overall distance from the reference set, and Spacing reflects the uniformity of the final point distribution. The weighted NSGA-II variants retain HV values in the range of 0.7360–0.7382, which remain comparable to MOPSO and slightly higher than SPEA2 and NSGA-III, while being much higher than MOEA/D. This indicates that the weighted search still spreads the obtained non-dominated solutions over a broad portion of the reference front. At the same time, within the NSGA-II variants, all three weighted schemes reduce GD relative to the unweighted baseline, from 0.0015 to 0.0009, 0.0006, and 0.0011, respectively. This indicates that the weighted search results lie closer to the global reference front, especially for the WSI-based and GSI-based formulations. The IGD values of the weighted variants remain clearly better than those of NSGA-III, MOPSO, and especially MOEA/D, although SPEA2 attains the smallest IGD. For Spacing, the weighted variants do not dominate uniformly, but the GLI-based scheme improves the distribution uniformity relative to the unweighted baseline, while SPEA2 gives the smallest overall Spacing. Therefore, the proposed weighting strategy improves not only how broadly the final solutions are distributed, but also how closely they approach the Pareto frontier, while maintaining competitive overall distribution quality. For completeness, full pairwise Pareto-front projections against NSGA-III, SPEA2, MOEA/D, and MOPSO are provided in Appendix A Figure A1.

**Figure 9:**
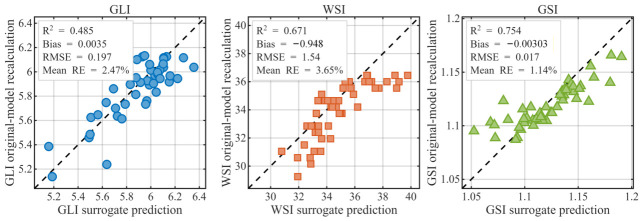
Local validation of candidate solutions in the Pareto region. The dashed line denotes the ideal relation y=x. The annotations report $R^{2}$, bias, RMSE, and mean relative error.

To further verify the final multi-objective optimization results, 50 Pareto-region points were selected for local validation. The MARS predictions were then compared with full theoretical-model recalculations, as shown in Figure 9. The mean relative errors are 2.47% for GLI, 3.65% for WSI, and 1.14% for GSI, indicating acceptable local prediction accuracy for engineering screening.

Although the global surrogate validation gives very high $R^{2}$ values, the Pareto-region samples are concentrated within a narrow advantageous band. In this local region, $R^{2}$ is sensitive to small differences among near-equivalent designs. Since the optimization is mainly used to identify an advantageous design region, RMSE and mean relative error are more relevant than fine pointwise ranking. Considering machining and assembly tolerances, the local errors are acceptable for engineering screening.

To select a final design for implementation, a compromise solution was chosen from the Pareto front. The primary selection criterion was the maximization of the GSI. A higher structural stiffness (GSI) directly translates to greater accuracy in load reproduction, as it minimizes undesirable deformations of the mechanism under load. Therefore, the solution exhibiting the highest GSI was identified from the front. A subsequent check confirmed that this design also maintains a significantly improved load-carrying capacity (GLI) and a substantial workspace (WSI), making it an excellent overall compromise.

The optimized design parameters are presented in $x_{\mathrm{opt}}$. To rigorously evaluate the effectiveness of the proposed optimization methodology, the performance of this final design is compared against a Baseline Design, whose parameters are presented in $x_{\mathrm{init}}$. This baseline represents a conventional, experience-based initial configuration, featuring symmetric hinge angles and a common platform radius ratio, serving as a meaningful benchmark for assessing improvement.

(28) $$\begin{array}{cc} x_{\mathrm{opt}} & ={\left[L_{\mathrm{rod}},\theta_{1},\theta_{2},r/R,\delta_{l}\right]}^{T}={\left[300.0,26.34,27.22,0.84,378.15\right]}^{T} \end{array}$$

(29) $$\begin{array}{cc} x_{\mathrm{init}} & ={\left[L_{\mathrm{rod}},\theta_{1},\theta_{2},r/R,\delta_{l}\right]}^{T}={\left[300.0,25.0,25.0,0.90,350.0\right]}^{T} \end{array}$$

A direct comparison of the performance indices, summarized in Table 4, demonstrates the substantial gains achieved by the optimization. The optimized design achieves a remarkable 27.1% improvement in load-carrying capacity (GLI). Concurrently, the Workspace Index (WSI) increased by 45.4%, and the Global Stiffness Index (GSI) improved by 15.4%.

These parameter shifts are consistent with the MARS-based sensitivity analysis reported above. The increase in $\delta_{l}$ from 350.0 mm to 378.15 mm is aligned with the dominant influence of stroke on WSI, while the decrease in r/R from 0.90 to 0.84 is consistent with the role of the platform aspect ratio in both workspace expansion and task-direction stiffness. The optimized values of $\theta_{1}$ and $\theta_{2}$ also depart from the symmetric baseline configuration, which agrees with their higher sensitivity to GLI and indicates that a modest redistribution of hinge angles improves force transmission efficiency. By contrast, $L_{\mathrm{rod}}$ remains at its lower bound under the present constraints, suggesting that the principal performance gains are realized mainly through the stroke, radius ratio, and hinge-angle adjustments.

**Figure 10:**
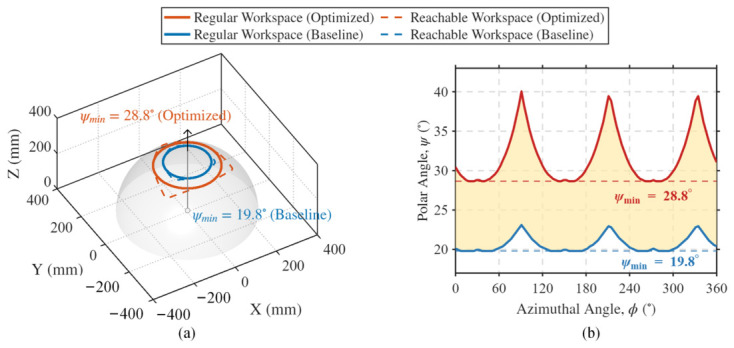

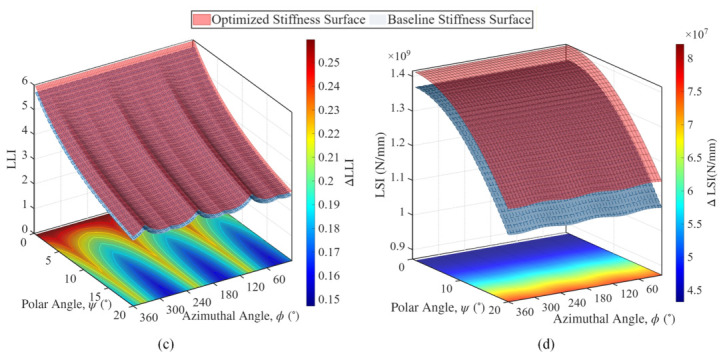
Workspace and local metrics for the optimized (red) and baseline (blue) designs. (**a**) 3D reachable and regular workspaces shown in Cartesian coordinates (X,Y,Z). (**b**) Workspace boundary profiles with polar angle ψ plotted against azimuthal angle ϕ; the shaded band indicates the workspace region and dashed horizontal lines mark $\psi_{\max}$ for each design. (**c**) Local Load capacity Index (LLI) surface over (ϕ,ψ) (vertical axis: LLI, dimensionless). (**d**) Local Stiffness Index (LSI) surface over (ϕ,ψ) (vertical axis: LSI in N/mm).

These global improvements are visually reported in Figure 10, which illustrates the enhanced local performance across the entire workspace. The optimized design (red surfaces) consistently outperforms the initial design (blue surfaces) in terms of the expanded workspace boundary (Figure 10a,b), the uniformly increased local stiffness (Figure 10c), and the significantly improved local load-carrying capacity (Figure 10d). The quantitative and qualitative results confirm the effectiveness of the proposed optimization framework in significantly enhancing the performance of the 6-UPS parallel mechanism.

## 4. Experiment

To physically verify the performance of the optimized loading system and to validate the effectiveness of the proposed optimization framework, an experimental platform was constructed and a series of validation tests were conducted.

### 4.1. Experimental Setup

**Figure 11:**
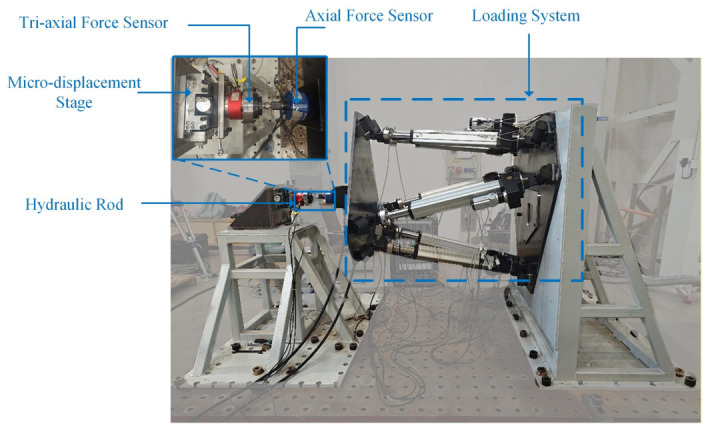
The force-loading experimental platform used to validate the optimized 6-UPS geometric configuration.

The experimental platform, shown in Figure 11, comprises two primary components:

1. Optimized 6-UPS Mechanism: The parallel mechanism was fabricated strictly according to the optimal geometric parameter set, $x_{\mathrm{opt}}$. The positioning system is driven by six fold-back electric linear actuators (YC80-T10-400-BR-FC-N20-P750), each powered by a high-response Panasonic servo motor (MHMF042L1C2M). This electromechanical configuration ensures high-stiffness and high-bandwidth orientation control for the moving platform.
2. Central Loading Actuator: The theoretical loading rod is replaced by a high-capacity servo-hydraulic cylinder. This actuator applies the primary vector force, while a tri-axial force sensor mounted at the end-effector provides ground-truth measurements of the applied load vector.

This hybrid arrangement employs the 6-UPS mechanism to define the spatial pose, while the central actuator applies the load magnitude. The purpose of this configuration is to evaluate the optimized geometric design under representative loaded poses while avoiding the additional complexity of full multi-axis active force coordination. In this setup, the 6-UPS mechanism is still required to sustain the reaction loads and maintain the commanded orientation, so the tests can verify the workspace-related pose feasibility, load-bearing stability, and vector-force reproduction capability of the optimized configuration under practical loading conditions. More specifically, this decoupled arrangement allows the geometry-dominated errors associated with pose generation, structural load transfer, and direction maintenance to be assessed separately from the additional errors introduced by coordinated limb-level force actuation. However, it should also be noted that this experimental platform is not intended as a complete validation of a fully active 6-UPS force-loading system. In particular, the present tests do not directly verify active torque suppression performance or multi-axis coupling errors arising from coordinated limb actuation. These aspects remain important topics for future experimental study. Similar scope-limited prototype validations are also common in parallel-manipulator design studies, where the optimized mechanism is first checked experimentally for feasibility or motion capability before full task-level realization is pursued [37,38].

### 4.2. Test Procedure

**Figure 12:**
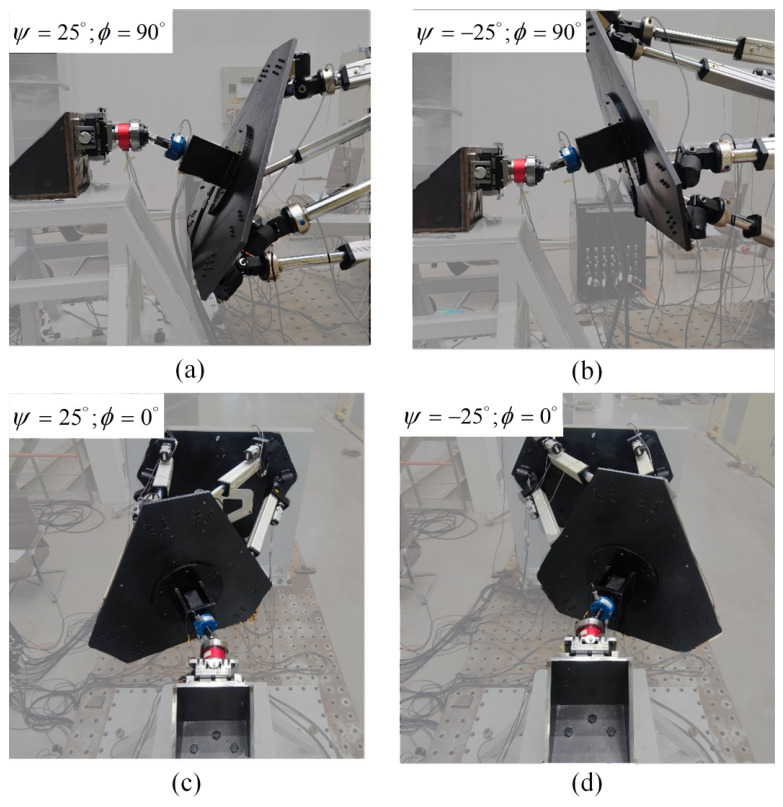
Experimental prototype of the 6-UPS mechanism at representative boundary orientations: (**a**) ψ=25°, ϕ=90°; (**b**) ψ=−25°, ϕ=90°; (**c**) ψ=25°, ϕ=0°; (**d**) ψ=−25°, ϕ=0°.

A full-factorial experimental protocol was designed to evaluate vector-force fidelity across the operational workspace shown in Figure 12. The test matrix encompasses 240 unique loading conditions, defined by permutations of the loading plane (azimuth ϕ), loading direction (polar angle ψ), and force magnitude (*F*):

- Loading Plane (azimuthal angle, ϕ): Two representative orthogonal planes were selected for testing: the horizontal plane (ϕ=0°) and the vertical plane (ϕ=90°).
- Loading Direction (polar angle, ψ): Within each plane, the polar angle of the force vector was varied from ψ=−25° to ψ=25° in steps of 5°.
- Force Magnitude: At each specified direction, the applied force was incrementally increased from 2 kN to 10 kN in steps of 2 kN.

Because the base and moving-platform joints are arranged with threefold rotational symmetry, the azimuthal behavior of the mechanism is periodic with a 120° interval, so the directional variation can in principle be reduced to a fundamental sector rather than the full 360° range [39]. Under the present experimental constraints, two orthogonal planes were then selected as characteristic sectional cuts within this symmetry-informed setting. Combined with the numerically predicted smooth azimuthal variation over the regular workspace, these tests provide representative sectional validation of the optimized workspace, but they should not be interpreted as exhaustive sampling of all azimuths. This interpretation is also consistent with prior regional-accuracy analyses of symmetric hexapod systems, where reduced workspace regions were found to preserve the overall accuracy trend [40].

During testing, the controller drove the moving platform to each target orientation and, after stabilization, the hydraulic rod applied the load sequence while a triaxial force sensor recorded the output force vector at every level. Within each plane, directions were scanned in a fixed order by sweeping ψ forward from 0° to 25°, and then backward from 25° to −25°. The same ordered sequence was then performed in the second plane. This four-segment schedule was then repeated once in the same order. In this way, each segment covered 6×5 conditions. Four segments formed one pass, and the entire pass was repeated twice. This deterministic routing of positioning, loading, and acquisition produced 240 completed test conditions.

### 4.3. Results and Analysis

**Figure 13:**
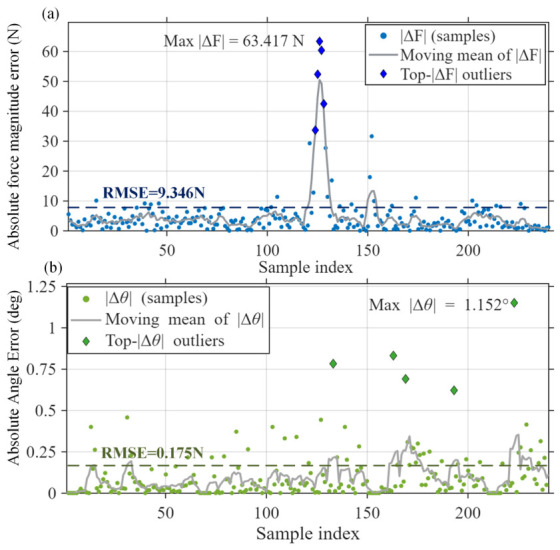
Experimental evaluation of the force-loading accuracy: (**a**) absolute force-magnitude error over all samples; (**b**) absolute force-direction error over all samples, with RMSE and maximum error values indicated.

The experimental performance, aggregated from all 240 test conditions, is summarized in Figure 13. As illustrated in Figure 13a, the system demonstrates high force tracking accuracy throughout the test campaign. The absolute magnitude error, |ΔF|, exhibits an RMSE of only 9.35 N. Even accounting for transient outliers, the maximum deviation remained below 63.42 N, representing less than 0.64% of the nominal 10 kN load. Under the adopted hybrid setup, this low error margin supports stable vector-force reproduction by the optimized pose-adjusting structure under practical loaded conditions.

Correspondingly, the angular alignment accuracy is presented in Figure 13b. The system maintained a directional error, |Δθ|, with an RMSE of 0.175°, where the maximum recorded angular deviation was 1.15° and the vast majority of samples remained below 1.0°. Crucially, the moving mean curves for both metrics remain essentially flat throughout the sample sequence. This stability indicates that the system’s high stiffness and accuracy are maintained consistently across the tested workspace without obvious degradation at the boundary poses.

The deterministic routing described in Section 4.2 also provides limited repeatability information because the same ordered pass was repeated twice. Identical conditions therefore recur with an offset of 120 samples. The major force-error burst around samples 120–140 and the isolated angular peak near sample 220 are not mirrored by equally strong peaks at the corresponding repeated conditions, which suggests that these events are more likely transient disturbances than systematic errors associated with a particular pose or load magnitude. This interpretation is consistent with the rapid recovery of the force error after the burst and with the absence of a synchronized spike in the angle-error curve.

At the same time, the present experimental evidence should be interpreted within its scope. The tests cover two representative azimuthal sections rather than the full three-dimensional workspace, and the hybrid setup does not directly verify fully active 6-UPS force coordination or torque suppression under coordinated limb actuation. Accordingly, the experiments validate the optimized geometry under loaded poses, but they do not constitute a complete experimental verification of a fully active multi-axis 6-UPS force-loading system.

## 5. Discussion

The results position the present work as a task-oriented engineering framework in which a 6-UPS vector-force loading mechanism is optimized by a bio-inspired evolutionary algorithm. Structurally, the main differentiating feature is the torque-decoupled loading path created by the central rod and specimen-side spherical joint. Algorithmically, the main contribution lies in assigning variable-wise distribution factors according to design-variable sensitivities, analogous to heterogeneous variation across constrained and weakly constrained loci in biological evolution. MARS serves as the sensitivity-estimation tool rather than the weighting principle itself. The comparisons in Figure 7 and Figure 8 indicate that this strategy improves convergence behavior and slightly reshapes the distribution of elite solutions, while the final Pareto boundary remains broadly similar across weighting choices. This reinforces the interpretation that the weighting mainly improves search efficiency and solution selection tendency for the present geometry-synthesis problem.

The term “design gene” is used here as a concise engineering analogy for parameter-level heterogeneity. It does not imply a direct biological model; rather, it explains why sensitivity information can be used to redistribute the search intensity among geometric variables.

The sensitivity results can also be interpreted from the viewpoint of phenotypic plasticity. In the present engineering analogy, the same geometric design vector can express different performance tendencies under different objective constraints. For example, actuator stroke mainly affects WSI, the platform radius ratio is closely related to GSI, and the hinge distribution angles are more influential for GLI. This objective-dependent behavior supports the use of variable-wise search intensities in the proposed NSGA-II strategy.

The experiments also need to be interpreted with appropriate boundaries. The hybrid platform provides useful evidence that the optimized geometry maintains the commanded pose and reproduces the target vector force with good accuracy under quasi-static loaded conditions. However, it does not directly verify fully active 6-UPS force coordination, active torque suppression, or multi-axis coupling errors under coordinated limb actuation. Likewise, the two tested azimuthal sections provide representative but not exhaustive coverage of the three-dimensional workspace. The analytical stiffness model should be viewed in the same way: it is a geometry-level approximation dominated by axial limb compliance, suitable for comparative optimization but not a replacement for a full elastostatic or finite-element model of the complete prototype.

Under the same computing environment, the surrogate-assisted framework reduces the wall-clock optimization cost from about 30 h for direct full-model NSGA-II optimization to about 3 h, including the one-time LHS sampling and MARS model-construction stage. From an application standpoint, this reduction transforms geometry synthesis from a bottleneck into a viable tool for rapid trade-off studies. Future work should therefore proceed in three directions: more extensive cross-family benchmarking under additional problem settings, more detailed structural validation using high-fidelity compliance models, and fully active experiments in which the 6-UPS limbs participate directly in force generation and residual-moment suppression.

## 6. Conclusions

This paper presented a task-specific 6-UPS loading system for aero-engine pylon vector-force loading and optimized its geometric configuration using a bio-inspired sensitivity-weighted NSGA-II framework. By using MARS to estimate design-variable sensitivities and embedding these sensitivities into the variable-wise search intensities of NSGA-II, the proposed methodology identified an optimized configuration that significantly outperforms the baseline, achieving improvements of 45.4%, 15.4%, and 27.1% in WSI, GSI, and GLI, respectively, while reducing optimization time by about 90%.

Prototype tests on a hybrid experimental platform further verified the optimized geometry under 240 quasi-static loaded pose conditions, yielding force magnitude errors below 0.64% and directional deviations below 1.15°. Within the scope of the adopted analytical model and hybrid experiments, these results support the proposed framework as an effective route for the geometry synthesis of high-fidelity aero-engine pylon static loading systems.

## Figures and Tables

**Table 1:** Global performance index fitting degree measurement index.

	GLI	WSI	GSI
MSE	1.078 × 10^−4^	0.202	9.708 × 10^−6^
$R^{2}$	0.998	0.995	0.998
GCV	4.586 × 10^−4^	4.572 × 10^−3^	1.176 × 10^−3^
NRMSE	4.392 × 10^−3^	1.498 × 10^−2^	7.789 × 10^−3^

**Table 2:** Comparison of the top-50 non-dominated-subset mean values under the four NSGA-II weighting schemes.

Weighting Scheme	${\mathrm{WSI}}_{\mathrm{top}50}$	${\mathrm{GSI}}_{\mathrm{top}50}$	${\mathrm{GLI}}_{\mathrm{top}50}$
Unweighted	34.929	1.1093	6.4168
GSI-based	35.396	1.1097	6.3984
WSI-based	35.423	1.1071	6.4127
GLI-based	35.352	1.1034	6.4233

**Table 3:** Normalized Pareto-quality indicators for the four NSGA-II weighting schemes and representative reference optimizers. The upward arrow indicates that a larger value is preferable, whereas the downward arrow indicates that a smaller value is preferable.

Method	HV↑	IGD↓	GD↓	Spacing↓
Unweighted NSGA-II	0.7396	0.0206	0.0015	0.0248
GSI-based NSGA-II	0.7360	0.0233	0.0009	0.0265
WSI-based NSGA-II	0.7382	0.0207	0.0006	0.0241
GLI-based NSGA-II	0.7381	0.0211	0.0011	0.0205
NSGA-III	0.7304	0.0310	0.0019	0.0244
SPEA2	0.7334	0.0177	0.0028	0.0113
MOEA/D	0.5891	0.2492	0.0005	0.0175
MOPSO	0.7386	0.0234	0.0010	0.0268

**Table 4:** Comparison of performance indices for the Baseline and Optimized designs.

Performance Index	Baseline Design	Optimized Design	Improvement
WSI (deg)	19.8	28.8	+45.4%
GSI	1.101	1.27	+15.4%
GLI	4.15	5.27	+27.1%

## Data Availability

The data presented in this study are available on request from the corresponding author.

## Appendix A. Additional Pareto-Front Projections Against Representative Multi-Objective Optimizers

To complement the within-framework comparison in Section 3.3, supplementary pairwise Pareto-front projections are provided in Figure A1. These appendix results compare the proposed NSGA-II-based weighting variants with representative reference optimizers, including NSGA-III, SPEA2, MOEA/D, and MOPSO. They are placed here to preserve the main mechanism-oriented narrative in the body text while still documenting the broader algorithmic context of the present problem.
